# *In silico* design of blue laser soft tissue vaporization with optimized optical power efficiency and mitigated thermal side effects

**DOI:** 10.1007/s10103-025-04514-8

**Published:** 2025-06-12

**Authors:** Takahiro Nishimura, Yusuke Watanabe, Yu Shimojo, Toshiyuki Ozawa, Daisuke Tsuruta

**Affiliations:** 1 Osaka Metropolitan University, Osaka, Japan; 2 The University of Osaka, Osaka, Japan; 3 Japan Society for the Promotion of Science, Tokyo, Japan

**Keywords:** Blue laser, Tissue vaporization, Coagulation, *In silico* evaluation

## Abstract

Blue diode laser irradiation has significant potential for realization of high vaporization efficiency with minimal thermal damage because of the strong blue light absorption of hemoglobin and the resulting shallow tissue penetration. This study presents an *in silico* framework for designing laser parameters, specifically pulse duration and power, for efficient vaporization under low-power irradiation conditions while minimizing thermal tissue damage. Computational simulations of laser-tissue interactions using the Monte Carlo light transport with dynamic optical properties model were conducted to evaluate vaporization and coagulation performance under various irradiation conditions. In addition to vaporization volume, the fraction of coagulated tissue was also calculated as a measure of thermal tissue damage. The *in silico* designs were validated experimentally through irradiation experiments performed on porcine liver tissue. Computational simulations revealed a non-monotonic relationship between pulse duration and vaporization volume at constant energy, as well as distinct trends for vaporization and coagulation. The experimental results confirmed the effectiveness of the simulation-derived parameters, and supported the practical utility of the proposed *in silico* design approach. The proposed *in silico* design approach enables quantitative analysis of vaporization and coagulation responses and can guide the development of safe and effective laser treatment protocols.

## Introduction

Clinical applications of blue diode lasers are gaining increasing interest because of their portability, cost-effectiveness, and ease of operation [1–3]. Blue laser irradiation offers higher vaporization efficiency and causes less thermal damage to the surrounding tissues than other lasers operating in the visible and infrared wavelength ranges [4–6]. This advantage is attributed to strong blue light absorption by hemoglobin and the shallower light penetration depth. Blue wavelength light also enables modulation of optical irradiation using various optical elements that are incompatible with CO$_2$ lasers and other infrared lasers [7]. This advanced control of blue laser light irradiation is expected to broaden the range of its applications in laser treatment. However, the irradiation power is limited by the damage thresholds of these optical systems [7, 8]. Efficient vaporization within the optical system’s power tolerance is thus essential.

**Fig. 1:**
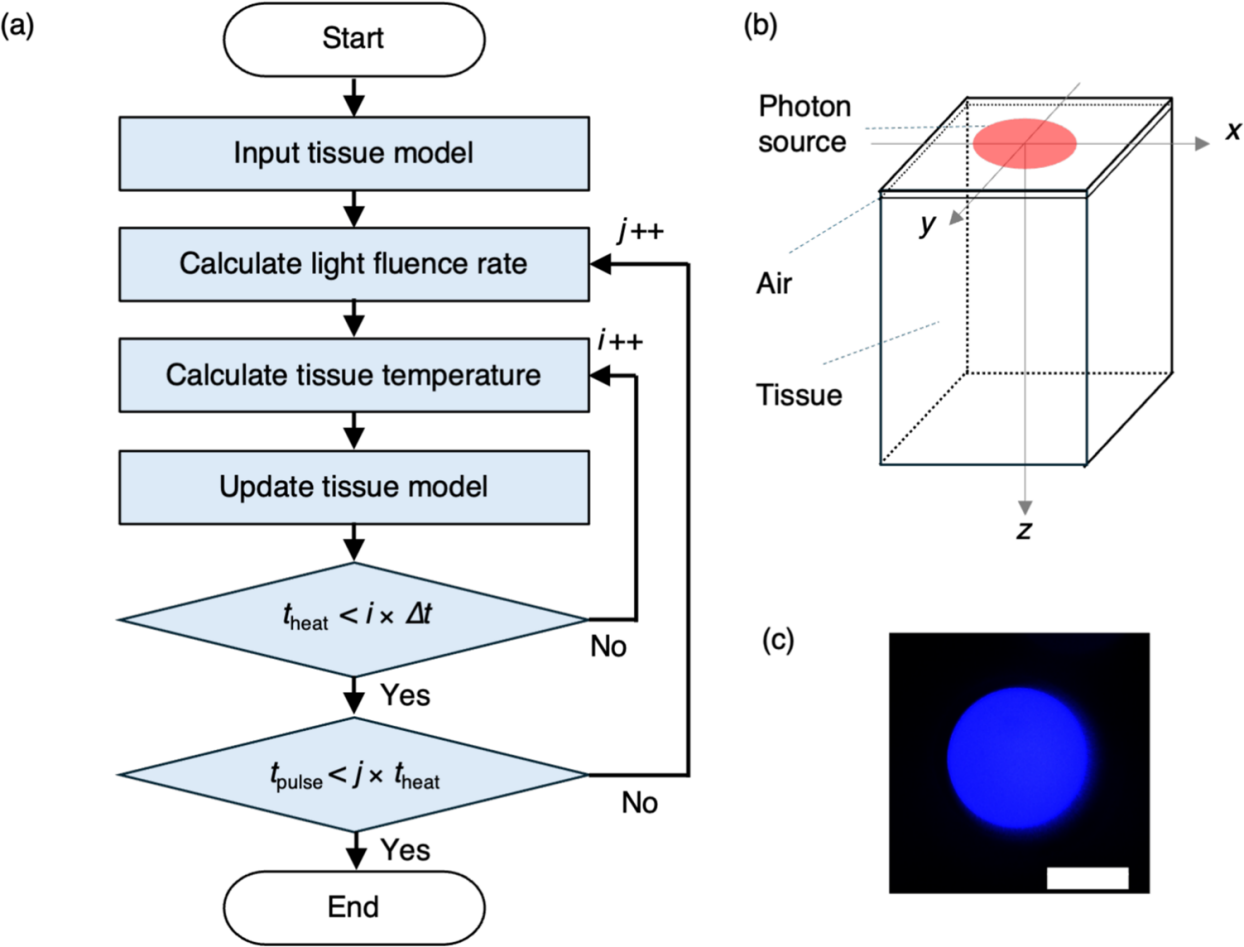
(a) Outline of the simulation flow. $\Delta t$, *i*, and $t_\textrm{heat}$ are the time step, iteration count, and calculation time for the thermal diffusion and thermal damage calculations, respectively. *j* and $t_\textrm{pulse}$ are the iteration count for the light transport calculations and the total calculation time, respectively. (b) Tissue geometry used in the numerical simulation, with coordinate axes and laser beam position. (c) Irradiated beam intensity distribution at a focal plane. The scale bar represents 200 $\mu$m

Laser vaporization of soft tissue has been performed clinically using various laser wavelengths [9]. For research and development of therapeutic applications, and for process evaluation, vaporization and coagulation measurements have been performed using both irradiated animal tissues [5] and tissue-mimicking phantoms [10]. The vaporization and coagulation outcomes depend not only on the optical properties of the tissues but also on the irradiation parameters, including the power and pulse duration [11]. Laser treatment device design requires consideration of a wide range of irradiation parameter combinations. One promising approach to this process is to perform *in silico* evaluations using computational simulations [12]. Several studies have applied finite element method (FEM)-based simulations to analyze coagulation in soft tissues under laser exposure [13, 14]. In addition, another method has been proposed to estimate vaporization and coagulation regions by incorporating dynamic changes in optical properties [15]. These methods estimate the vaporization and coagulation regions accurately by modeling dynamic changes in the optical properties and simulating the light distribution and thermal damage within tissues.

One promising approach to this process is to perform *in silico* evaluations using computational simulations [12, 15]. Recently, three-dimensional simulation methods for tissue vaporization and coagulation have been reported [16, 17]. These methods estimate the vaporization and coagulation regions accurately by modeling dynamic changes in the optical properties and simulating the light distribution and thermal damage within tissues. This *in silico* evaluation approach based on numerical simulation methods enables efficient design of irradiation parameters through numerical evaluation of the optical irradiation conditions.

This study presents an *in silico* design of the irradiation conditions for low-power blue laser vaporization. The relationship between the pulse duration and the photothermal effects in blue diode lasers is evaluated to determine optimal pulse durations for enhanced vaporization efficiency and reduced thermal damage under constrained irradiation power conditions. Previous studies have introduced a computational simulation method for laser vaporization based on the Monte Carlo (MC) light transport using dynamic optical properties (DOP) model [17]. Although the simulation method was originally developed for 980 nm infrared diode lasers, the present study extends its application to blue diode lasers (445 nm) by substituting wavelength-specific optical and thermal parameters. We assume that the core physical processes, such as light propagation, absorption, photothermal conversion, heat diffusion, and vaporization, are consistent across wavelengths, and thus the same computational structure can be applied with updated input parameters. In this study, a DOP-based vaporization simulation is used to assess the secondary coagulation effects that occur during laser vaporization quantitatively and then establish an irradiation protocol that can mitigate these effects. This *in silico* evaluation approach enables quantitative assessment by eliminating biological variability and reducing the validation time significantly. The validity of this *in silico* design is verified experimentally using porcine tissue, thus confirming the effectiveness and efficiency of the proposed design method.

**Table 1:** Parameters for air and tissue layers

Parameter	Air	Tissue
Scattering coefficient $$\mu _\textrm{s}$$ (mm$$^{-1}$$)	0.001	18.1$$^*$$/49.4$$^{**}$$
Absorption coefficient $\mu _\textrm{a}$ (mm$^{-1}$)	0.0	1.1
Anisotropy factor *g* (-)	1.0	0.9
Refractive index *n* (-)	1.0	1.4
Thermal conductivity *k* (W/(cm$\cdot$K))	$2.602 \times 10^{-4}$	$5.43 \times 10^{-3}$
Specific heat capacity $c_p$ (J/(g$\cdot$K))	1.006	3.542
Density $\rho$ (g/cm$^3$)	$1.196 \times 10^{-3}$	1.0
Frequency factor *Z* (1/s)	-	$7.39 \times 10^{37}$
Activation energy $E_a$ (J/mol)	-	$2.577 \times 10^5$
Gas constant *R* (J/(mol$\cdot$K))	-	8.314
Vaporization temperature $T_v$ ($^\circ$C)	-	100
Latent heat of vaporization $L_v$ (J/g)	-	$2.257 \times 10^3$

$$^*$$native, $$^{**}$$coagulation

## Material and methods

### Mathematical model

Figure 1(a) shows an outline of the numerical simulation flow for laser-induced tissue vaporization and coagulation. The detailed methodology for this simulation has been reported previously in Ref. [17]. First, a three-dimensional numerical model called $M(\mathrm{\textbf{r}}, t)$ ($\mathrm{\textbf{r}}$: position vector in tissue; *t*: time) was prepared that consists of an air layer and a tissue layer. The optical and thermal properties specific to the air and tissue layers were individually assigned to each layer. Then, the absorption distribution $A(\mathrm{\textbf{r}}, t)$ resulting from laser irradiation to $M(\mathrm{\textbf{r}}, t)$ was calculated using a Monte Carlo light transport simulation, based on the assigned optical properties. By assuming that all absorbed light energy was converted into thermal energy, the heat source distribution was then derived from $A(\mathrm{\textbf{r}}, t)$. This assumption neglects potential photochemical effects, such as those arising from molecular excitation at 445 nm. However, previous studies have not reported significant photochemical tissue damage under comparable irradiation conditions [18]. Therefore, the simplifying assumption that all absorbed energy is converted into heat was adopted in this study to focus on thermally driven vaporization and coagulation processes. This assumption may result in overestimation of thermal effects, particularly in vaporization and coagulation extent. The enthalpy $H(\mathrm{\textbf{r}}, t)$ and temperature $T(\mathrm{\textbf{r}}, t)$ distributions were calculated at each time step $\Delta t$ using the heat diffusion equation. From the calculated $T(\mathrm{\textbf{r}}, t)$ distribution, the thermal damage parameter distribution $\Omega (\mathrm{\textbf{r}}, t)$ was calculated using the Arrhenius model. Regions where $\Omega (\mathrm{\textbf{r}}, t) > 1$, i.e., where it exceeded the coagulation threshold, were updated in $M(\mathrm{\textbf{r}}, t)$ to represent the coagulated tissue layer. Then, regions in which $H(\mathrm{\textbf{r}}, t)$ exceeded the vaporization threshold were updated to become part of the air layer. On the basis of these updated areas in $M(\mathrm{\textbf{r}}, t)$, the optical and thermal properties were also updated. This calculation step was repeated until the total time exceeded the calculation time for thermal diffusion and thermal damage with little change in $\Omega (\mathrm{\textbf{r}}, t)$ during light transport, $t_\textrm{heat}$. Following the update of $M(\mathrm{\textbf{r}}, t)$, the absorption energy distribution $A(\mathrm{\textbf{r}}, t)$ was recalculated, with subsequent calculations and updates of $H(\mathrm{\textbf{r}}, t)$, $T(\mathrm{\textbf{r}}, t)$, and $M(\mathrm{\textbf{r}}, t)$. This procedure was repeated until the time exceeded the laser pulse duration $t_\textrm{pulse}$. After the laser irradiation time ended, $A(\mathrm{\textbf{r}}, t)$ was set at zero, and the iterative calculations and updates of $H(\mathrm{\textbf{r}}, t)$, $T(\mathrm{\textbf{r}}, t)$, and $M(\mathrm{\textbf{r}}, t)$ were repeated until the temperature decreased to a level at which it no longer affected the tissue.

### Numerical tissue model

Figure 1(b) shows the initial state $M(\mathrm{\textbf{r}}, 0)$ used in the experiment. The voxel size was set at $0.02 \, \text {mm} \times 0.02 \, \text {mm} \times 0.02 \, \text {mm}$, with a voxel count of $150 \times 150 \times 200$. Along the *z*-axis, the air layer extended from 0 mm to 0.1 mm, and the tissue layer then spanned from 0.1 mm to 4.0 mm. The tissue layer was modeled based on the properties of porcine liver to align with the experimental conditions. The optical and thermal properties of the air and tissue layers are listed in Table 1. The optical properties at the wavelength of 450 nm were taken from measured porcine liver data given in Ref [17]. The optical and thermal properties of the coagulation area were set to have the same values as the tissue layer, apart from the scattering coefficient. The scattering coefficient of tissue region $\mu _\textrm{s}'(\mathrm{\textbf{r}}, t)$ was varied based on the thermal damage. For simplicity during calculations, the tissue was modeled as a binary system composed of native and coagulated tissues. Although tissue damage in reality progresses continuously [17], we approximated it using a binary tissue damage model, in which each voxel was assumed to be either native or coagulated based on a thermal damage threshold.

### Light transport simulation

The absorption distribution $A(\mathrm{\textbf{r}}, t)$ was calculated using a three-dimensional MC simulation code, Monte Carlo eXtrem (MCX) [19]. This simulation accounts for multiple scattering events within tissue by using the scattering coefficient $\mu _\textrm{s}$ and anisotropy factor *g* corresponding to the laser wavelength. The optical properties of porcine liver tissue at a wavelength of 450 nm reported in Ref [17] were used (see Table 1). The laser beam was centered at (*x*, *y*) = (0 mm, 0 mm) and propagated along the *z*-axis to irradiate the tissue surface vertically. The beam profile had a uniform distribution with a diameter of 350 $\mu$m, reflecting the experimentally observed irradiance shape (see Fig. 1(c)). A uniform distribution was therefore selected in the simulation to match the actual beam profile. The number of photon packets was set at $10^7$ per Monte Carlo simulation. This value was determined with reference to previous Monte Carlo studies [17], considering the trade-off between computational cost and statistical accuracy. The pulse width and the irradiation power were adjusted according to the specified conditions. In the simulations, the incident laser power was varied from 0.1 W to 200 W, and the pulse duration was varied from 10 ms to 4000 ms. The optical penetration depth *d*, defined as the distance at which the light intensity along the optical axis decreases to 1/*e*, was 0.28 mm in the Monte Carlo simulation. It should be noted that this simulation does not account for the interaction between the incident laser and vaporized material or plasma that may form above the tissue surface during vaporization. Secondary effects such as beam attenuation or scattering caused by the vapor plume are neglected. This simplification was adopted to focus on subsurface energy deposition and thermal effects within the tissue.

### Thermal diffusion and damage simulation

$H(\mathrm{\textbf{r}}, t)$ and $T(\mathrm{\textbf{r}}, t)$ were calculated by solving the following enthalpy method equation using the finite difference method [17].

1 $$\begin{aligned} \frac{\partial H(\mathrm{\textbf{r}}, t)}{\partial t} = k \nabla ^2 T(\mathrm{\textbf{r}}, t) + A(\mathrm{\textbf{r}}, t), \end{aligned}$$

where $k$ represents the thermal conductivity. The relationship between $T(\mathrm{\textbf{r}}, t)$ and $H(\mathrm{\textbf{r}}, t)$ is given as follows:

2 $$\begin{aligned} T(\mathrm{\textbf{r}}, t) = {\left\{ \begin{array}{ll} \frac{H(\mathrm{\textbf{r}}, t)}{\rho c_\textrm{p}} &  \text {for } H(\mathrm{\textbf{r}}, t)< \rho c_\textrm{p}T_\textrm{vap},\\ T_\textrm{vap} &  \text {for } \rho c_\textrm{p} T_\textrm{vap}< H(\mathrm{\textbf{r}}, t) < \rho (c_\textrm{p} T_\textrm{vap} + L_\textrm{vap}), \end{array}\right. } \end{aligned}$$

where $\rho$, $c_p$, $T_{\text {vap}}$, and $L_{\text {vap}}$ are the density (g/cm$^3$), the specific heat capacity (J/(g$\cdot$K)), the vaporization temperature ($^\circ$C), and the latent heat of vaporization (J/g), respectively. In this study, the finite difference method (FDM) was employed for thermal diffusion modeling due to its simplicity and compatibility with voxel-based Cartesian grids used in MC simulations. While the FEM can support more complex geometries and coupled thermal-mechanical analysis, the present work focused solely on thermal response and vaporization efficiency. The initial air and tissue temperatures were set at room temperature (22 $^\circ$C). To evaluate the coagulation region, the Arrhenius damage parameter $\Omega (\mathrm{\textbf{r}}, t)$ was calculated as follows:

3 $$\begin{aligned} \Omega (\mathrm{\textbf{r}}, t) = Z \int _0^{t} \exp \left( -\frac{E_a}{RT(\mathrm{\textbf{r}}, t)} \right) \, \textrm{d}t \end{aligned}$$

where *Z*, $E_a$, and *R* are the frequency factor (1/s), the activation energy (J/mol), and the gas constant (J/(mol$\cdot$K)), respectively. A voxel was classified as coagulated region if it satisfied $\Omega (\mathrm{\textbf{r}}, t) \ge 1$ without exceeding a vaporization threshold temperature. In this study, tissue vaporization was modeled by assuming that the water content in the tissue is in a liquid state prior to vaporization. Accordingly, only the latent heat of vaporization was considered in the energy balance. The latent heat of melting was not included, as the tissue water is assumed to be liquid under physiological conditions. Tissue vaporization was assumed to follow the process of water evaporation. The enthalpy $H_{\text {th}}$ required for vaporization is defined as the sum of the heat required to raise the temperature to boiling point and the latent heat of vaporization:

4 $$\begin{aligned} H_{\text {th}} = \rho (c_\textrm{p} T_\textrm{vap} + L_\textrm{vap}) \end{aligned}$$

where $C_p$ is the specific heat, $T_{\text {vap}}$ is the vaporization temperature, and $L_{\text {vap}}$ is the latent heat of tissue vaporization. Regions where $H(\mathrm{\textbf{r}}, t) > H_{\text {th}}$ were classified as ablated regions, where the tissue was removed and changed into air. The simulation parameters are listed in Table 1. $\Delta t$ was set at 0.01 ms and $t_\textrm{heat}$ was set at 5 ms. This value was selected based on previous studies [17], where $t_\textrm{heat}$ corresponds to the typical cavitation period in liquid.

The tissue’s thermal relaxation time $\tau$ was calculated using the following equation.

5 $$\begin{aligned} \tau = \frac{d^2 \rho c_p}{4 k}, \end{aligned}$$

where *d*, $\rho$, $c_p$, and $k$ represent the optical penetration depth (mm), density (g/cm$^3$), specific heat capacity (J/(g$\cdot$K)), and thermal conductivity (W/(cm$\cdot$K)), respectively. Here, *d* was defined as the axial distance at which the light intensity falls to 1/*e* of its surface value, and was calculated to be 0.28 mm using the Monte Carlo light transport simulation.

**Fig. 2:**
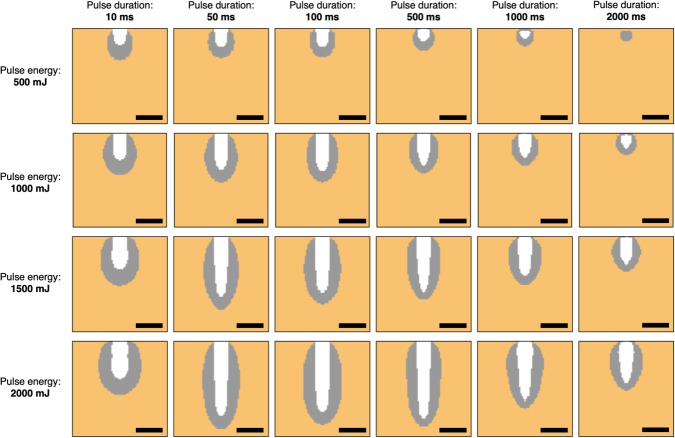
Vaporization simulation results for various pulse durations (10, 50, 100, 500, 1000, and 2000 ms) under constant pulse energy conditions (500, 1000, 1500, and 2000 mJ). The *yz*-planes at *x* = 0 are shown. The tops represent the air-tissue boundaries under the initial conditions. The orange regions represent the tissue areas, the white regions represent the vaporization areas, and the gray regions indicate the coagulation areas. The scale bars represent 500 $\mu$m

### Laser irradiation experiment

A fiber-coupled laser diode system (DS3, BWT Beijing) was used to perform the irradiation experiments. A continuous wave at a wavelength of 445 ± 10 nm was guided through an optical fiber with a core diameter of 200 $\mu$m and a numerical aperture (NA) of 0.22. The laser light emitted from the fiber was then collimated using an aspherical lens with a focal length of 18 mm (ACL2018U, Thorlabs) and focused onto the sample surface using a spherical lens with a 30 mm focal length (LA1805-AB, Thorlabs). The sample was placed on a z-axis translation stage (TAR-34802, Sigma Koki) and positioned to focus the laser on the sample’s surface plane. The light intensity distribution on the focal plane was captured using a complementary metal-oxide-semiconductor (CMOS) camera to measure the beam diameters. The light intensity distribution was disk-like in shape and had an almost constant intensity (Fig. 1(b)). The diameter was 350 $\mu$m. The measured Rayleigh length was 2 mm. The irradiation power was measured using a thermal sensor (FL250A-BB-35, Ophir). The pulse duration and irradiation power were adjusted according to the required experimental conditions.

### Sample preparation

Porcine liver tissue (Tokyo Shibaura Zouki) samples were prepared. The tissue was frozen and sliced to a thickness of 10 mm using a meat slicer (PMS-220F, Minato Works). The samples were then cut to fit inside a petri dish and left to thaw at room temperature (22°C) for 2 h. During the thawing process, the tissue samples were sealed with paraffin to prevent drying.

### Measurement of vaporization and coagulation

After laser irradiation, the samples were immersed in a 10% formalin solution (Fujifilm Wako Pure Chemical Corporation) at 4 ${}^\circ$C for 24 h. Each sample has been subsequently washed three times in 0.01 mol/L phosphate-buffered saline (PBS; Fujifilm Wako Pure Chemical Corporation), with each wash lasting 10 min. Using a cryostat (CM1850, Leica), the samples were sectioned through the center of their irradiation sites. Cross-sections of the tissue slices were imaged using a CMOS camera, and the vaporized and coagulated regions were segmented using ImageJ (full details are given in Ref [16]). The vaporization and coagulation areas were then calculated.

**Fig. 3:**
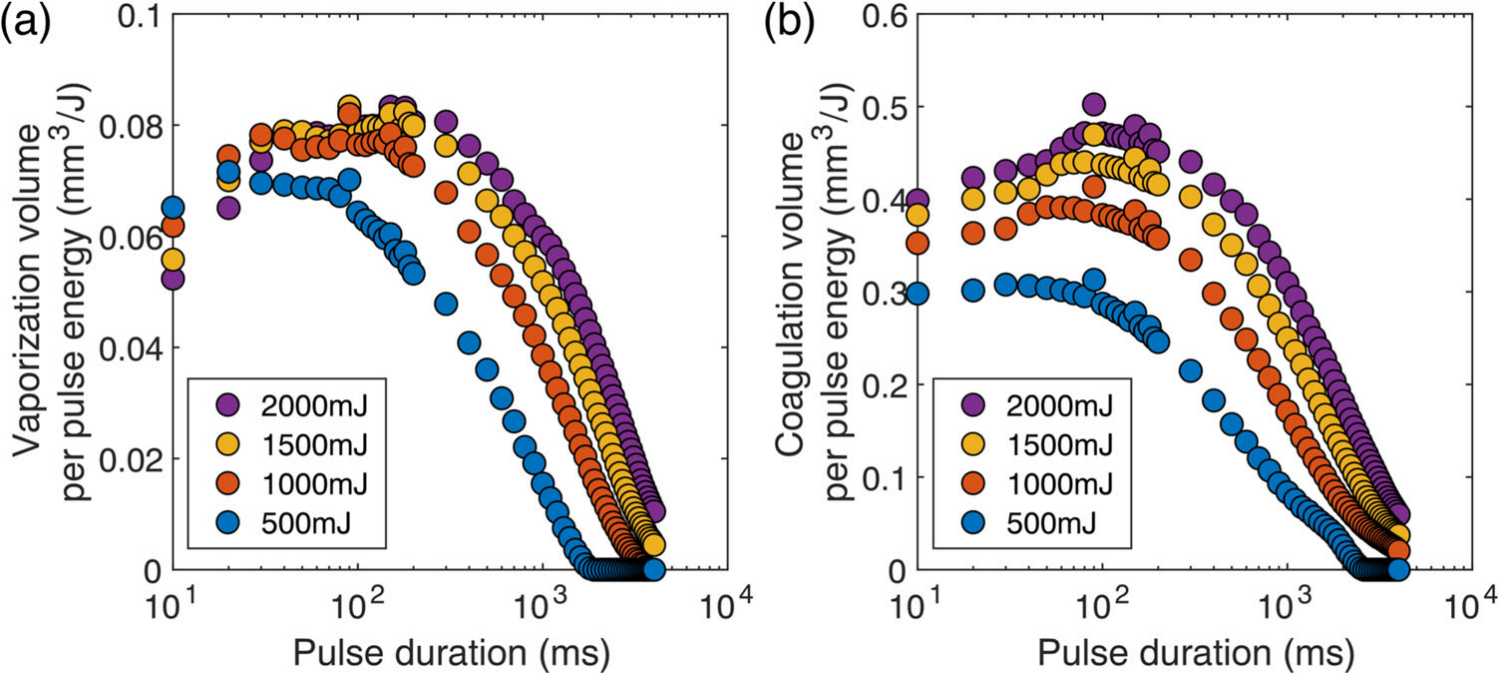
(a) Vaporization and (b) coagulation volumes per unit pulse energy versus pulse duration

## Results

### Distributions of vaporization and coagulation zones

Figure 2 shows the cross-sectional simulation results for various pulse energies and pulse durations. When simulations with the same pulse width are compared, an increase in the pulse energy correlated with a greater vaporization depth. Conversely, at the same pulse energy, the vaporization depth reached a maximum when the pulse duration was 50, 100, or 500 ms. This corresponded to durations that were roughly equal to or exceeded the thermal relaxation time (1.3 $\times$ 10$^2$ ms). Although the thermal relaxation time is not explicitly included as a parameter in the simulation model, its effects are inherently captured through the heat diffusion process. In our model, the spatial and temporal evolution of temperature is governed by the heat diffusion equation based on tissue-specific thermal properties such as thermal conductivity, density, and specific heat. Because the model dynamically simulates temperature propagation following laser absorption, the role of thermal relaxation in determining vaporization and coagulation behavior is effectively incorporated. Furthermore, when the pulse duration is very short (e.g., less than 50 ms), although thermal confinement is achieved, vaporization efficiency decreases due to the mismatch with the thermal response time of the tissue. In this model, the characteristic thermal interaction time ($t_{\textrm{heat}}$) is assumed to be 5 ms, corresponding to the cavitation period of liquid [20]. If energy exceeding the vaporization threshold is deposited in a time shorter than $t_{\textrm{heat}}$, vaporization may not proceed fully, and excess energy is dissipated as non-specific heating, resulting in lower vaporization efficiency. Under low pulse energy conditions (500 mJ), zero vaporization occurred when the pulse duration was extended to 2000 ms. The coagulation zone was observed to form around the vaporization zone, with its extent increasing to match the size of the vaporization zone. However, the distance from the deepest point in the vaporization zone to the deepest point in the coagulation zone decreased as the pulse duration increased. These findings indicate that the vaporization volume and the coagulation zone are influenced significantly by the pulse energy and duration settings. The results suggest that pulse energy and duration optimization is essential to enhance the vaporization efficiency while also minimizing coagulation-related damage.

### Vaporization and coagulation efficiencies versus incident energy

To determine vaporization conditions that are efficient and cause minimal damage relative to the incident energy, the vaporization efficiency and coagulation efficiency were evaluated versus the incident energy. Here, the vaporization volume and the coagulation volume per unit incident energy were defined as the vaporization energy efficiency and the coagulation energy efficiency, respectively, and used to perform a comparative evaluation. Figure 3(a) shows the relationship between the pulse duration and the vaporization energy efficiency. Although the vaporization energy efficiency was generally lower at 500 mJ when compared with the other conditions, it reached an upper limit of approximately 0.08 mm$^3$/J at 1000 mJ, 1500 mJ, and 2000 mJ. This suggests that vaporization energy efficiency is determined primarily by the pulse duration and cannot be improved by altering the power settings. Furthermore, at all pulse energies, the vaporization energy efficiency declined sharply beyond a pulse duration threshold of 10$^2$–10$^3$ ms. These findings indicate that it is essential to maintain the pulse duration within this range for sustained vaporization energy efficiency. Figure 3(b) shows the relationship between the pulse duration and the coagulation energy efficiency. Unlike the vaporization energy efficiency, which showed minimal variation with the incident energy, the coagulation energy efficiency tended to increase as the incident energy rose. This suggests that, within the energy range studied here, the coagulation energy efficiency is dependent on power when the pulse duration remains constant.

**Fig. 4:**
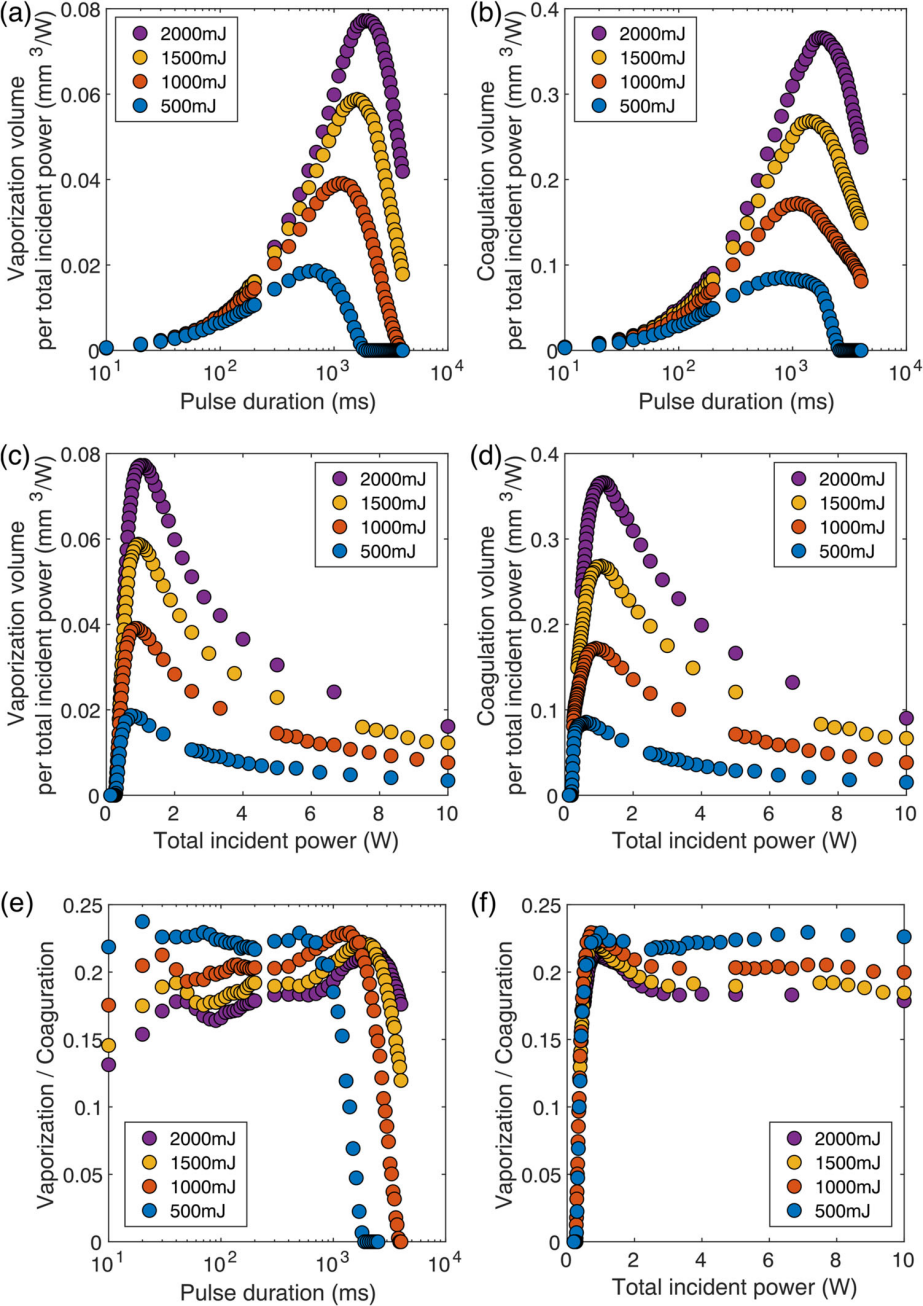
(a) Vaporization and (b) coagulation volumes per unit total incident power versus pulse duration. (c) Vaporization and (d) coagulation volumes per unit total incident power versus total incident power. Vaporization to coagulation volume ratio per unit total incident power versus (e) pulse duration and (f) total incident power

### Vaporization and coagulation efficiencies versus incident power

In the long-pulse irradiation case, the optical system’s damage threshold is often limited by power rather than energy. To evaluate the conditions under which the damage threshold is restricted by power, the vaporization power efficiency and the coagulation power efficiency were assessed. The vaporization and coagulation volumes per unit power were defined as the vaporization power efficiency and the coagulation power efficiency, respectively. Figure 4(a) shows the relationship between the pulse duration and the vaporization power efficiency. In all cases, a peak vaporization power efficiency was observed at a pulse duration of approximately 1000 ms. Additionally, higher pulse energies corresponded to greater vaporization power efficiencies. Figure 4(b) shows the relationship between the coagulation power efficiency and the pulse duration. The coagulation power efficiency also varied with the irradiation power. When compared with the vaporization efficiency, it showed steeper rises at shorter pulse durations before reaching a peak and then gradually declining. Figure 4(c) and (d) show the irradiation power’s relationships with the vaporization power efficiency and the coagulation power efficiency, respectively. Both the vaporization power and the coagulation power efficiencies peaked within the 0.5–2 W range, but the peak power for the coagulation power efficiency shifted toward higher values. These findings illustrate that the vaporization power efficiency and the coagulation power efficiency do not follow identical trends. This suggests that it may be possible to identify conditions that suppress the coagulation power efficiency while maintaining the vaporization power efficiency. This selective optimization is clinically significant because excessive coagulation can lead to increased collateral thermal damage and prolonged healing times, whereas efficient vaporization enables precise tissue removal. In soft tissue laser surgery, minimizing unnecessary coagulation while achieving effective vaporization improves treatment accuracy and safety [12, 21].

**Fig. 5:**
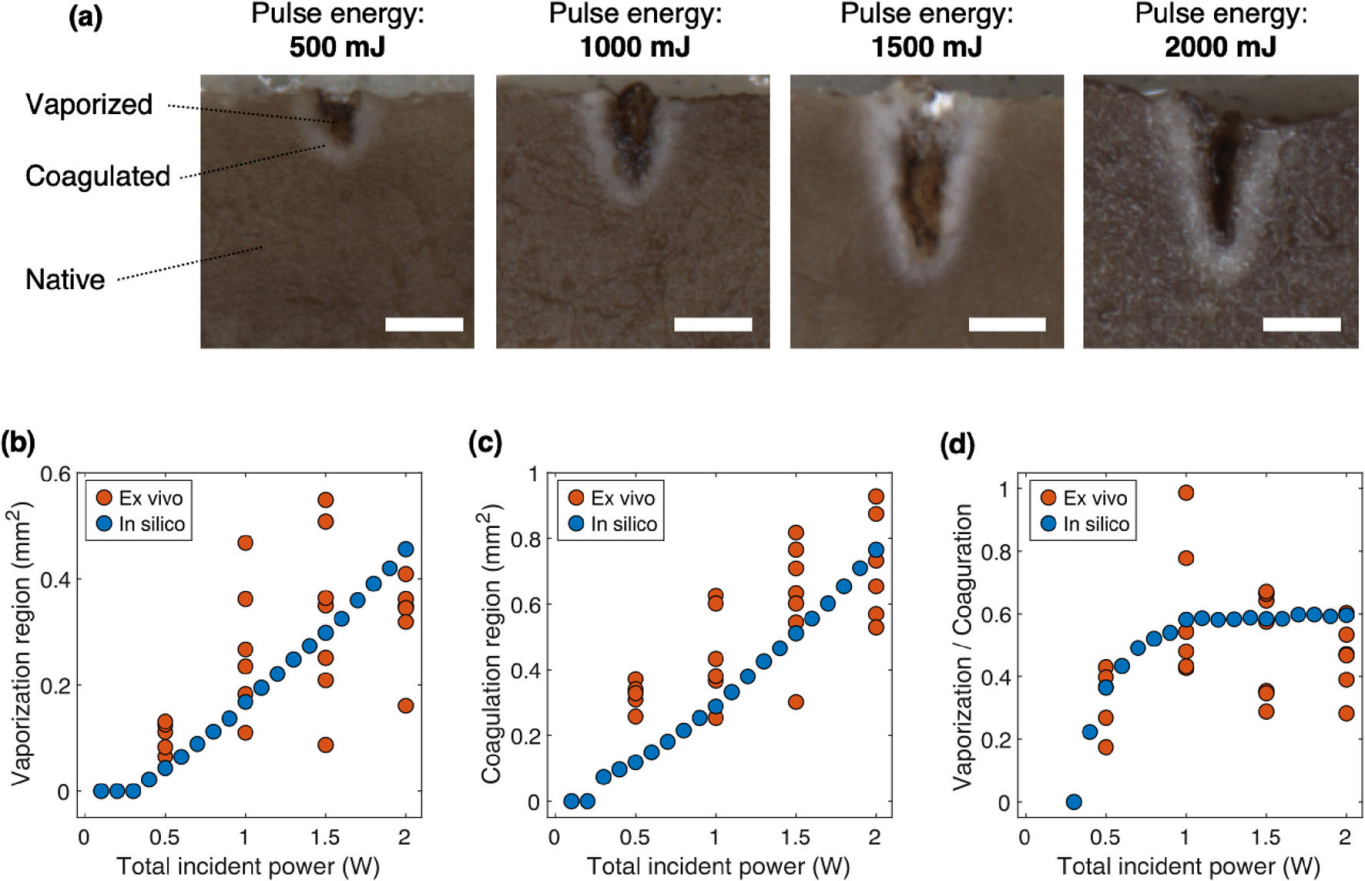
(a) Tissue cross-section after laser irradiation. Pulse duration was fixed at 1000 ms, while the laser power was varied. The scale bars represent 500 $\mu$m. Experimental (*ex vivo*) and numerical (*in silico*) results for (b) vaporization and (c) coagulation areas versus total incident power. (d) Experimental (*ex vivo*) and numerical (*in silico*) results for vaporization to coagulation volume ratio versus total incident power

### Comparison of coagulation to vaporization ratio

Figures 3 and 4(a)-(d) revealed that changes in the irradiation conditions in the vaporization and coagulation regions do not necessarily align. To determine conditions that suppress the power coagulation efficiency without compromising the power vaporization efficiency, the ratio of the vaporization volume to the coagulation volume was analyzed. Figure 4(e) illustrates the vaporization-to-coagulation ratio as a function of the pulse duration. When the pulse duration exceeded 1000 ms, this ratio decreased, regardless of the irradiation energy. To suppress coagulation effectively, it is necessary to set the pulse duration to be less than 1000 ms; however, the suppression effect was found to be minimal, even at shorter pulse durations. Furthermore, at durations exceeding 1000 ms, an increase in the irradiation energy resulted in a further reduction in the vaporization-to-coagulation ratio, indicating that longer pulse durations produce increased coagulation volumes. Figure 4(f) shows the vaporization-to-coagulation ratio as a function of the irradiation power. Specifically, the ratio rose sharply within the 0.5–1 W range and then plateaued at a constant value. Although irradiation above approximately 1 W is required to suppress coagulation effectively, these results indicate that increasing the vaporization power beyond 1 W yields only marginal coagulation suppression benefits.

### Experimental validation

The *in silico* analysis indicated that setting a pulse duration of less than 1000 ms would achieve tissue vaporization without compromising the coagulation ratio (Fig. 4(e)). To validate the simulation results that indicate that 1 W irradiation is sufficient for coagulation suppression, the vaporization-to-coagulation ratio was evaluated experimentally by varying the power levels for a fixed irradiation time of 1000 ms. Figure 5(a) shows cross-sectional images of the tissue after irradiation. When the irradiation power increased, both the vaporization and coagulation regions expanded. Figure 5(b), (c), and (d) compare the simulation results with the experimental values. Although the experimental variability was significant, the trend observed at 1 W, where the coagulation ratio increased, was consistent with the *in silico* design. Although the vaporization efficiency decreased at pulse durations of less than 1000 ms, the experiments confirmed the predicted outcomes. It was verified that a maximum pulse duration of 1000 ms combined with 1 W irradiation minimizes the coagulation ratio while maintaining vaporization. These results confirm the validity of the irradiation conditions designed via numerical simulations and provide reliable guidelines for obtaining optimal settings.

## Discussion

During low-power blue laser vaporization, this study demonstrated that design of the pulse duration and the irradiation power effectively improved the vaporization efficiency while suppressing coagulation. Understanding the influence of the incident energy and pulse duration time settings on the vaporization and coagulation efficiencies provides essential guidance for selection of treatment irradiation conditions in future applications. Moreover, comparison of simulated and experimental results indicates that simulation-based determination of the optimal conditions is viable in practice.

The simulation results revealed that vaporization power efficiency does not increase monotonically with laser power or pulse duration. As shown in Fig. 4(e,f), the efficiency peaked at an intermediate pulse duration of approximately 1000 ms and moderate power levels between 1.0 and 1.5 W. These results indicate that excessive power or extremely short or long pulse durations may lead to lower vaporization efficiency, possibly due to insufficient thermal diffusion. This non-monotonic behavior highlights the importance of identifying irradiation conditions that balance vaporization performance and thermal damage, rather than simply maximizing power or energy. The results also suggest that careful tuning of pulse duration and power can enhance treatment efficiency under practical low-power constraints, as required in clinical endoscopic systems.

Although laser fluence is commonly used as a threshold parameter to characterize vaporization efficiency, particularly in short-pulse or high-peak-power regimes [22], it may not fully capture the vaporization behavior observed under long-pulse, low-power conditions. In our study, thermal diffusion and pulse duration significantly influence the spatial and temporal evolution of tissue heating, meaning that vaporization is not solely determined by fluence. Therefore, while fluence-based analyses are valuable, they were not the focus of this work.

In this study, the optical properties of porcine liver were used. The comparison between the irradiation experiments and the simulation results (Fig. 5(b-d)) showed consistent trends, with identified conditions yielding similar outcomes. However, the experimental results showed significant variability, which was attributed to individual differences and heterogeneity in the physical parameters. Here, the individual differences refer to sample-to-sample variations in optical and thermal properties, such as those arising from biological variability between different porcine liver samples. The heterogeneity refers to spatial variations within a single sample, including localized differences in absorption, scattering, and thermal diffusion properties. This underscores the difficulty of deriving optimal conditions through experimental analysis alone. *In silico* design, however, provides comparisons under completely identical conditions. Nevertheless, incorporating variability in the physical parameters into *in silico* designs remains a challenge for future research. To address individual differences, methods including probabilistic modeling using virtual patients, as proposed in the in silico clinical trial approach, have been proposed [23]. Developing similar techniques to represent parameter variability will be a critical task for future advancement of laser treatments.

The photothermal effects were evaluated for single pulses with varying pulse durations without considering repetition of the laser pulses. When the pulse interval is short, insufficient tissue layer cooling can lead to heat accumulation, thus increasing the thermal damage area. Empirically, a pulse interval of at least ten times the thermal relaxation time is recommended to allow adequate tissue cooling [24]. For stationary irradiation, repeated pulse irradiation must occur at intervals greater than this threshold to avoid increased thermal damage. Additionally, using a beam scanner to disperse the beam spatially can prevent heat accumulation. Under these conditions, the irradiation settings at the pulse durations established in this study remain effective.

## Conclusion

This study presented an *in silico* approach for designing of optimal pulse duration and irradiation power in blue diode laser vaporization under low-power conditions. By coupling Monte Carlo light transport with dynamic optical properties and thermal diffusion modeling, we quantitatively evaluated both vaporization and coagulation outcomes. The simulation results revealed a non-monotonic relationship between pulse duration and vaporization efficiency, highlighting the importance of balancing thermal confinement and energy deposition dynamics. Experimental validation using porcine liver tissue confirmed the simulation-predicted trends, demonstrating the practical utility of the proposed simulation-guided design. These findings support the feasibility of using simulation-based methods to design safe and effective laser treatment protocols, especially under constrained power conditions. The results also underscore the potential of blue diode lasers for precise, thermally efficient vaporization in endoscopic laser therapies.

## Data Availability

Data and simulation codes provided within the manuscript are available from the corresponding author on reasonable request.

## Appendix: List of nomenclature

**Table 2:** List of nomenclature

Symbol	Description
$A(\textbf{r}, t)$	Absorbed energy distribution
$M(\textbf{r}, t)$	Tissue type distribution
$T(\textbf{r}, t)$	Temperature distribution
$H(\textbf{r}, t)$	Enthalpy distribution
$\Omega (\textbf{r}, t)$	Arrhenius thermal damage distribution
$\mu _a$	Absorption coefficient
$\mu _s$	Scattering coefficient
*g*	Anisotropy factor
*n*	Refractive index
*k*	Thermal conductivity
$c_p$	Specific heat capacity
$\rho$	Density
*Z*	Frequency factor
$E_a$	Activation energy
*R*	Gas constant
$T_{\text {vap}}$	Vaporization temperature
$L_{\text {vap}}$	Latent heat of vaporization
$L_m$	Latent heat of melting
$\Delta t$	Time step
$t_{\text {heat}}$	Thermal interaction time
$t_{\text {pulse}}$	Laser pulse duration
$\tau$	Thermal relaxation time
*d*	Optical penetration depth
